# A systematic review of speech recognition technology in health care

**DOI:** 10.1186/1472-6947-14-94

**Published:** 2014-10-28

**Authors:** Maree Johnson, Samuel Lapkin, Vanessa Long, Paula Sanchez, Hanna Suominen, Jim Basilakis, Linda Dawson

**Affiliations:** Faculty of Health Sciences, Australian Catholic University, 40 Edward Street, 2060 North Sydney, NSW Australia; Centre for Applied Nursing Research (a joint facility of the South Western Sydney Local Health District and the University of Western Sydney), Affiliated with the Ingham Institute of Applied Medical Research, Sydney, Australia; Central Queensland University, Bundaberg, Australia; University of Western Sydney, Sydney, Australia; Department of Information Technology, NICTA, The Australian National University, College of Engineering and Computer Science, University of Canberra, Faculty of Health, and University of Turku, Canberra, ACT Australia; University of Wollongong, Wollongong, Australia

**Keywords:** Nursing, Systematic review, Speech recognition, Interactive voice response systems, Human transcriptions, Health professionals

## Abstract

**Background:**

To undertake a systematic review of existing literature relating to speech recognition technology and its application within health care.

**Methods:**

A systematic review of existing literature from 2000 was undertaken. Inclusion criteria were: all papers that referred to speech recognition (SR) in health care settings, used by health professionals (allied health, medicine, nursing, technical or support staff), with an evaluation or patient or staff outcomes. Experimental and non-experimental designs were considered.

Six databases (Ebscohost including CINAHL, EMBASE, MEDLINE including the Cochrane Database of Systematic Reviews, OVID Technologies, PreMED-LINE, PsycINFO) were searched by a qualified health librarian trained in systematic review searches initially capturing 1,730 references. Fourteen studies met the inclusion criteria and were retained.

**Results:**

The heterogeneity of the studies made comparative analysis and synthesis of the data challenging resulting in a narrative presentation of the results. SR, although not as accurate as human transcription, does deliver reduced turnaround times for reporting and cost-effective reporting, although equivocal evidence of improved workflow processes.

**Conclusions:**

SR systems have substantial benefits and should be considered in light of the cost and selection of the SR system, training requirements, length of the transcription task, potential use of macros and templates, the presence of accented voices or experienced and in-experienced typists, and workflow patterns.

## Background

### Introduction

Technologies focusing on the generation, presentation and application of clinical information in healthcare, referred to as health informatics or eHealth solutions [1, 2] have experienced substantial growth over the past 40 years. Pioneering studies relating to technologies for producing and using written or spoken text, known as computational linguistics, natural language processing, human language technologies, or text mining, were published in the 1970s and 1980s [3–10]. Highlights of the 1990s and early 2000s include the MedLEE Medical Language Extraction and Encoding System to parse patient records and map them to a coded medical ontology [11] and the Autocoder system to generate medical diagnosis codes from a patient record [12]. Today, a literature search using Pubmed for computational linguistics, natural language processing, human language technologies, or text mining recovers over 20,000 references.

Health informatics or eHealth solutions enable clinical data to become potentially accessible through computer networks for the purposes of improving health outcomes for patients and creating efficiencies for health professionals [13–16]. Language technologies hold the potential for making information easier to understand and access [17].

Speech recognition, in particular, presents some interesting applications. Speech recognition (SR) systems compose of microphones (converting sound into electrical signals), sound cards (that digitalise the electrical signals) and speech engine software (that convert the data into text words) [18]. As early as 1975 speech recognition systems were described ‘in which isolated words, spoken by a designed talker, are recognized through calculation of a minimum prediction residual’ [19] reporting a 97.3 per cent recognition rate for a male speaker. Applications have been demonstrated in radiology [20] with the authors noting a reduction in turnaround time of reports from 15.7 hours to 4.7 hours, although some difficulties with integration of systems have also been identified [21]. Document processing within endocrinology and psychiatry including physicians and their secretaries also demonstrated improvements in productivity [22]. Similar approaches have recently been applied in the reporting of surgical pathology with improvements in ‘turnaround time from 4 to 3 days’ and ‘cases signed out in 1 day improved from 22% to 37%’ [23]. These authors also alluded to the issue of correction of errors and the use of templates [23] for processing of information.

Although systematic reviews of health informatics [24–27] have been conducted, surprisingly we were unable to locate such a review on speech recognition in health care.

### Aim

The aim of this study was to undertake a systematic review of existing literature relating to SR applications, including the identification of the range of systems, implementation or training requirements, accuracy of information transfer, patient outcomes, and staff considerations. This review will inform all health professionals about the possible opportunities and challenges this technology offers.

## Methods

All discoverable studies published in the refereed literature from the year 2000 and in English language only were included in the review. We believed that only studies from 2000 onwards would use speech recognition technology that was sufficiently accurate to be suitable for health care settings. Papers were included if they referred to speech recognition in health care settings, being used by health professionals (allied health, medicine, nursing, technical or support staff), with an evaluation of patient or staff outcomes. All research designs, experimental and non-experimental, were included. Studies were excluded if they were opinion papers or describing technical aspects of a system without evaluation. Methods for searching the literature, inclusion criteria, and general appraisal and analysis approaches were specified in advance in an unregistered review protocol.

### Data sources (Search strategy)

Six databases (Ebscohost including CINAHL, EMBASE, MEDLINE including the Cochrane Database of Systematic Reviews, OVID Technologies, PreMED-LINE, PsycINFO) were searched by a qualified health librarian trained in systematic review searches, using the following search terms: “automatic speech recognition”, “Speech Recognition Software”, “interactive voice response systems”, “((voice or speech) adj (recogni* or respon*)).tw.”, “(qualitative* or quantitative* or mixed method* or descriptive* or research*).tw.”. It should be noted that EMBASE includes 1000 conference proceedings (grey material) also. In addition, a search was undertaken for grey literature in Open Grey. Examples of the searches undertaken from three major databases are presented in Table 1.

**Table 1:** **Search strategies OVID Embase, Medline, PreMedline**

**OVID Embase**		
1	automatic speech recognition/	469
2	((voice or speech) adj (recogni* or respon*)).tw.	2516
3	or/1-2	27490
4	exp research/	380483
5	(qualitative* or quantitative* or mixed method* or descriptive* or research*).tw.	1194784
6	or/4-5	14148120
7	3 and 6	483
8	limit 7 to yr = “2000 -Current”	433
**OVID Medline**		
1	Speech Recognition Software	416
2	((voice or speech) adj (recogni* or respon*)).tw.	2081
3	or/1-2	2263
4	exp Research/	224487
5	(qualitative* or quantitative* or mixed method* or descriptive* or research*).tw.	840821
6	or/4-5	971456
7	3 and 6	360
8	limit 7 to yr = “2000 -Current”	319
**OVID PreMedline**		
1	((voice or speech) adj (recogni* or respon*)).tw.	140
2	(qualitative* or quantitative* or mixed method* or descriptive* or research*).tw.	94513
3	1 and 2	20
4	limit 3 to yr = “2000 -Current”	19

Note that Speech Recognition Software refers to a MeSH term. * = wildcard.

### Selection of studies

**Figure 1:**
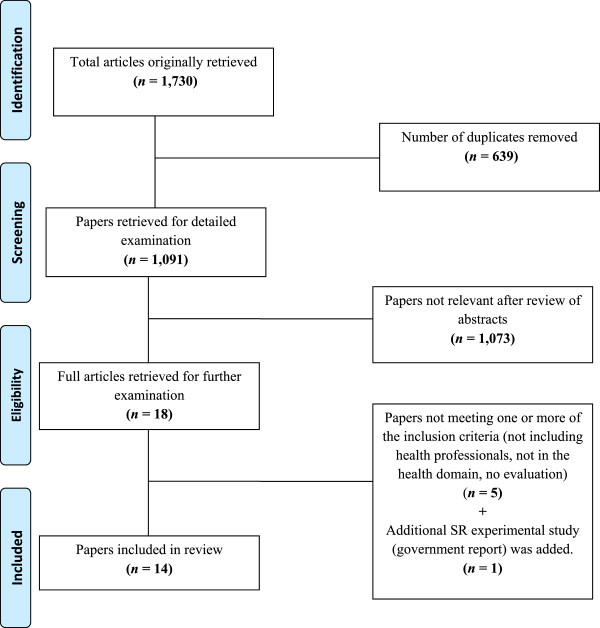
**Selection of studies for the review.**

The search identified 1,730 references to publications that were published in or after 2000. There were 639 duplicates in these 1,730 references which were removed resulting in 1,091. Some 1,073 papers were not found to be relevant as they reflected other topics or applications such as: auditory research (65), cochlear implant or hearing instrument (174), conversations, or multiple speakers (12), discrete speech utterance (2), impaired voice (150), informal research notes including comments or response (6), interactive voice response (199), speech perception (53), synthesized speech (4), thesis (1), and other irrelevant topics (340). The remaining 18 were examined using the inclusion criteria by two independent reviewers and 14 papers (see Figure 1) were retained. All identified abstracts were reviewed by two reviewers, and a third where there was disagreement. The relevant full text of the article was obtained and then if the paper met the eligibility criteria (checked by two reviewers) the study was included. Inclusion criteria were: referred to speech recognition in health care settings, used by health professionals (allied health, medicine, nursing, technical or support staff), with evaluation of patient or staff outcomes.

The quality of each eligible study was rated by two independent reviewers using the Mixed Methods Appraisal Tool (including a range of quantitative designs the focus in this review) [28]. The scores for the included studies ranged from 4 to 6 out of a possible maximum of 6 [22, 29] (See Table 2). Data were extracted from the relevant papers using a specifically designed data extraction tool and due to the nature of the content reviewed by two reviewers.

**Table 2:** **SR Quality scoring of included studies - Mixed Methods Appraisal Tool (MMAT)-Version 2011**

	Al-Aynati 2003 [18]	Alapetite, 2008 [30]	Alapetite, 2009 [31]	Callaway, 2002 [20]	Derman, 2010 [32]	Devine, 2000 [33]	Irwin, 2007 [34]	Kanal, 2001 [35]	Koivikko, 2008 [36]	Langer, 2002 [37]	Mohr, 2003 [22]	NSLHD 2012 [29]	Singh, 2011 [23]	Zick, 2001 [38]
**Screening Questions**														
Clear research questions	Yes	Yes	Yes	Yes	Yes	Yes	Yes	Yes	Yes	Yes	Yes	Yes	Yes	Yes
Appropriate data collected	Yes	Yes	Yes	Yes	Yes	Yes	Yes	Yes	Yes	Yes	Yes	Yes	Yes	Yes
**1. Qualitative**														
Appropriate qualitative data sources														
Appropriate qualitative method														
Description of the context														
Discussion of researchers’ reflexivity														
**2. Randomized controlled**														
Appropriate randomization											Yes	No		
Allocation concealment and/or blinding											Yes	No		
Complete outcome data											Yes	Yes		
Low withdrawal/drop out											Yes	Yes		
**Screening Questions**														
**3. Non-randomized**														
Recruitment minimized bias	No													
Appropriate outcome measures	Yes													
Intervention & control group comparable	Yes													
Complete outcome data/acceptable response rate	Yes													
**4. Quantitative descriptive**														
Appropriate sampling^1^		No	Yes	Yes	No	Yes	Yes	No	Yes	Yes			Yes	No
Appropriate sample^2^		No	Yes	Yes	Yes	Yes	Yes	Yes	Yes	Yes			Yes	Yes
Appropriate measurement (valid/standard)		Yes	Yes	Yes	Yes	Yes	Yes	Yes	Yes	Yes			Yes	Yes
Acceptable response rate		Yes	Yes	Yes	Yes	Yes	No	Yes	Yes	No			Yes	Yes
**Total Score** ^**3**^ **(Yes =1, No = 0)**	**5**	**4**	**6**	**6**	**5**	**6**	**5**	**5**	**6**	**5**	**6**	**4**	**6**	**5**

^1^Sampling strategy relevant to address the quantitative research question. Consider whether the source of sample is relevant to the population under study; when appropriate, there is a standard procedure for sampling; the sample size is justified (using power calculation for example).

^2^Sample representative of the population under study. Consider whether inclusion and exclusion criteria are explained; reasons why certain eligible individuals chose not to participate are explained.

^3^Scores ranged from 0–6.

### Description and methodological quality of included studies

Of the fourteen studies retrieved, one was a randomised controlled trial (RCT) [22]; ten were comparative experimental studies [18, 20, 23, 29, 32–34, 36–38] and most of the remaining were descriptive studies predominately using a survey design [30, 31, 35].

The studies were conducted in hospitals or other clinical settings including: emergency departments [29, 38], endocrinology [22]; mental health [22, 32], pathology [18, 23], radiology [20, 35–37]; and dentistry [34]. However, one study was carried out in a laboratory setting simulating an operating room [30].

The health professionals or support staff involved were: nurses [29], pathologists [23], physicians [22, 29, 31, 32, 38], radiologists [18, 35, 36], secretaries [22], transcriptionists [18, 22] and undergraduate dental students [34]. In one study no participants were identified [30].

Training varied between studies with some studies providing data based on minimal training 5 minutes [29] to 30 minutes [23] to 6 hours [22]. One study emphasised the need for one to two months use before staff were familiar with SR [32].

The majority of the papers focused on systems that supported English language, however other languages such as Finnish [36] and Danish [30] were also investigated. Participants in two studies were non native English speakers although they transcribed documents into English [18, 35].

The quality scores for the studies ranged from two studies at 4 [29, 30], six studies at 5 [18, 32, 34, 35, 37, 38], and six studies at 6 [20, 22, 23, 31, 33, 36], with 6 being the maximum score possible (see Table 2).

### Outcomes of the studies

The main outcome measures in the included studies were: productivity including report turnaround time [20, 22, 23, 29, 36–38]; and accuracy [18, 22, 29, 38].

The findings of the included studies were heterogeneous in nature, with diverse outcome measures, which resulted in a narrative presentation of the studies (See Table 3).

**Table 3:** **Summary of speech recognition (SR) review results**

Author	Aim	Setting	Outcome measures	Results
Year		Sample		
Country Design			Speech technology (ST)	
Design				
Al-Aynati and Chorneyko 2003 [18]	To compare SR software with HT for generating pathology reports	**Setting:** Surgical pathology	1. Accuracy rate	**Accuracy rate (mean %)**
		**Sample:** 206 pathology reports	2. Recognition/ Transcription errors	SR: 93.6 HT: 99.6
Canada Experimental		**ST:** IBM Via Voice Pro version 8 with pathology vocabulary dictionary		**Mean recognition errors**
				SR: 6.7 HT: 0.4
Mohr et al. 2003 [22]	To compare SR software with HT for clinical notes	**Setting:** Endocrinology and Psychiatry	1. Dictation/recording time + transcription (minutes) = Report Turnaround Time (RTT).	**RTT (mins)**
				**Endocrinology**
				SR: (Recording + transcription) = 23.7
				HT: (Dictation + transcription) = 25.4
USA Experimental		**Sample:** 2,354 reports		
		**ST:** Linguistic Technology Systems LTI with clinical notes application		SR: 87.3% (CI 83.3, 92.3) productive compared to HT.
				**Psychiatry transcriptionist**
				SR: (Recording + transcription) = 65.2
				HT: (Dictation + transcription) = 38.1
				SR: 63.3% (CI 54.0, 74.0) productive compared to HT.
				**Psychiatry secretaries**
				SR: (Recording + transcription) = 36.5
				HT: (Dictation + transcription) = 30.5
				SR: 55.8% (CI 44.6, 68.0) productive compared to HT.
				Author, secretary, type of notes were predictors of productivity (p < 0.05).
NSLHD 2012 [29]	To compare accuracy and time between SR software and HT to produce emergency department reports	**Setting:** Emergency Department	1. RTT	**RTT mean (range) in minutes**
Australian Experimental		**Sample:** 12 reports		SR: 1.07 (46 sec, 1.32)
		**ST:** Nuance Dragon Voice Recognition		HT: 3.32 (2.45, 4.35)
				HT: Spelling and punctuation errors
				SR: Occasional misplaced words
Alapetite, 2008 [30]	To evaluate the impact of background	**Setting:** Simulation laboratory	1. Word Recognition Rate (WRR)	**WRR**
Denmark Non-experimental	noise (sounds of alarms, aspiration, metal, people talking, scratch, silence, ventilators) and other factors affecting SR accuracy when used in operating rooms	**Sample:** 3600 short anaesthesia commands		**Microphone**
				Microphone 1: Headset 83.2%
		**ST:** Philips Speech Magic 5.1.529 SP3 and Speech Magic Inter Active Danish language, Danish medical dictation adapted by Max Manus		Microphone 2: Handset 73.9%
				**Recognition mode**
				Command 81.6%
				Free text 77.1%
				**Background noise**
				Scratch 66.4%
				Silence 86.8%
				**Gender**
				Male 76.8%
				Female 80.3%
Alapetite et al. 2009 [31]	To identify physician’s perceptions, attitudes and expectations of SR technology.	**Setting:** Hospital (various clinical settings)	1. Users’ expectation and experience	**Overall**
Denmark Non-experimental		**Sample**: 186 physicians	Predominant response noted.	**Q1** Expectation: positive 44%
				**Q1** Experience: negative 46%
				**Performance**
				**Q8** Expectation: negative 64%
				**Q8** Experience: negative 77%
				**Time**
				**Q14** Expectation: negative 85%
				**Q14** Experience: negative 95%
				**Social influence**
				**Q6** Expectation negative 54%
				**Q6** Experienced negative 59%
Callaway et al. 2002 [20]	To compare an off the shelf SR software with manual transcription services for radiology reports	**Setting:** 3 military medical facilities	1. RTT (referred to as TAT)	**RTT**
USA Non-experimental		**Sample:** Facility 1: 2042 reports	2. Costs	**Facility 1:** Decreased from 15.7 hours (HT) to 4.7 hours (SR)
		Facility 2: 26600 reports		**Completed in <8 h:** SR 25% HT 6.8%
		Facility 3: 5109 reports		**Facility 2:** Decreased from 89 hours (HT) to 19 hours (SR)
		**ST:** Dragon Medical		**Cost**
		Professional 4.0		**Facility 2:** $42,000 saved
				**Facility 3:** $10,650 saved
Derman et al. 2010 [32]	To compare SR with existing methods of data entry for the creation of electronic progress notes	**Setting:** Mental health hospital	1. Perceived usability	**Usability**
Canada Non-experimental		**Sample:** 12 mental health physicians **ST:** Details not provided	2. Perceived time savings	50% prefer SR
			3. Perceived impact	**Time savings:** No sig diff (p = 0.19)
				**Impact**
				**Quality of care** No sig diff (p = 0.086)
				**Documentation** No sig diff (p = 0.375)
				**Workflow** No sig improvement (p = 0.59)
Devine et al. 2000 [33]	To compare ‘out-of-box’ performance of 3 continuous SR software packages for the generation of medical reports.	**Sample:** 12 physicians from Veterans Affairs facilities New England	1. Recognition errors (mean error rate)	**Recognition errors (mean-%)**
USA Non-experimental		**ST: System 1 (S1)** IBM ViaVoice98 General Medicine Vocabulary.	2. Dictation time	**Vocabulary**
			3. Completion time	**S1** (7.0 -9.1%) **S3** (13.4-15.1%) **S2** (14.1-15.2%)
		**System 2 (S2)** Dragon Naturally Speaking Medical Suite, V 3.0.	4. Ranking	**S1** Best with general English and medical abbreviations.
				**Dictation time:** No sig diff (P < 0.336).
		**System 3 (S3)** L&H Voice Xpress for Medicine, General Medicine Edition, V 1.2.	5. Preference	**Completion time (mean):**
				**S2** (12.2 min) **S1** (14.7 min) **S3** (16.1 min)
				**Ranking: 1** **S1** **2** **S2** **3** **S3**
Irwin et al. 2007 [34]	To compare SR features and functionality of 4 dental software application systems.	**Setting:** Simulated dental	1. Training time	**Training time**
USA Non-experimental		**Sample:** 4 participants (3 students, 1 faculty member)	2. Charting time	**S1** 11 min 8 sec **S2** 9 min 1 sec (no data reported for **S3** ad **S4**).
			3. Completion	
		**ST: Systems 1 (S1)** Microsoft SR with Dragon NaturallySpeaking.	4. Ranking	**Charting time: S1** 5 min 20 sec **S2** 9 min 13 sec, (no data reported for **S3** ad **S4**).
		**System 2 (S2)** Microsoft SR		**Completion %: S1** 100 **S2** 93 **S3** 90 **S4** 82
		**Systems 3 (S3) & System 4 (S4)** Default speech engine.		**Ranking**
				**1 S1** 104/189 **2 S2** 77/189
Kanal et al. 2001 [35]	To determine the accuracy of continuous SR for transcribing radiology reports	**Setting:** Radiology department	1. Error rates	**Error rates (mean ± %)**
USA Non-experimental		**Sample:** 72 radiology reports 6 participants		**Overall** (10.3 ± 33%)
				**Significant errors** (7.8 ± 3.4%)
		**ST:** IBM MedSpeaker/Radiology software version 1.1		**Subtle significant errors** (1.2 ± 1.6%)
Koivikko et al. 2008 [36]	To evaluate the effect of speech recognition onadiology workflow systems over a period of 2 years	**Setting:** Radiology department	1. RTT (referred to as TAT) at 3 collection points:	**RTT (mean ± SD) in minutes**
Finland Non-experimental		**Sample: >**20000 reports; 14 Radiologists	HT: 2005 (n = 6037)	HT: 1486 ± 4591
		**ST:** Finnish Radiology Speech	SR_1_: 2006 (n = 6486)	SR_**1:**_ 323 ± 1662
		Recognition System (Philips Electronics)	SR_2_: 2007 (n = 9072)	SR_**2**_: 280 ± 763
		HT: cassette-based reporting	2. Reports completed ≤ 1 hour	**Reports ≤ 1 hour (%)**
		SR1: SR in 2006		HT: 26
		SR2: SR in 2007		SR_**1**_: 58
		**Training:**		
		10-15 minutes training in SR		
Langer 2002 [37]	To compare impact of SR on radiologist productivity. Comparison of 4 workflow systems	**Setting:** Radiology departments	1. RTT (referred to as TAT)	**RTT (mean ± SD%) in hours/ RP**
USA Non-experimental		**Sample:** Over 40 radiology sites	2. Report productivity (RP), number of reports per day	**System 1**
		**System 1** Film, report dictated, HT		RTT: 48.2 ± 50 RP: 240
		**System 2** Film, report dictated, SR		**System 2**
		**System 3** Picture archiving and communication system + HT		RTT: 15.5 ± 93 RP: 311
				**System 3**
		**System 4** Picture archiving and communication system + SR		RTT: 13.3 ± 119 (t value at 10%) RP: 248
				**System 4**
				RTT: 15.7 ± 98 (t value at 10%) RP: 310
Singh et al. 2011 [23]	To compare accuracy and turnaround	**Setting:** Surgical pathology	1. RTT (referred to as TAT)	**RTT in days**
USA Non-experimental	times between SR software and traditional transcription service (TS) when used for generating surgical pathology reports	**Sample:** 5011 pathology reports	2. Reports completed ≤ 1 day	**Phase 0:** 4
		**ST:** VoiceOver (version 4.1) Dragon Naturally Speaking Software (version 10)	3. Reports completed ≤ 2 day	**Phase 1:** 4
			**Phase 0:** 3 years prior SR	**Phase 2–4:** 3
			**Phase 1:** First 35 months of SR use, gross descriptions	**Reports ≤ 1 day (%)**
				Phase 0: 22
			**Phase 2–4:** During use of SR for gross descriptions and final diagnosis	Phase 1: 24
				Phase 2–4: 36
				**Reports ≤ 2 day (%)**
				Phase 0: 54
				Phase 1: 60
				Phase 2–4: 67
Zick et al. 2001 [38]	To compare accuracy and RTT between	**Setting:** Emergency Department	1. RTT (referred to as TAT)	**RTT in mins**
USA Non-experimental	SR software and traditional transcription service (TS) when used for recording in patients’ charts in ED	**Sample:** Two physicians - 47 patients’ charts	2. Accuracy	SR: 3.55 TS: 39.6
			3. Errors per chart	**Accuracy % (Mean and range)**
		**ST:** Dragon NaturallySpeaking Medical suite version 4	4. Dictation and editing time	SR: 98.5 (98.2-98.9) TS: 99.7 (99.6-99.8)
			4. Throughput	**Average errors/chart**
				SR: 2.5 (2–3) TS: 1.2 (0.9-1.5)
				**Average dictation time in mins (Mean and range)**
				SR: 3.65 (3.35-3.95) TS: 3.77 (3.43-4.10)
				**Throughput (words/minute)**
				SR: 54.5 (49.6-59.4) TS: 14.1 (11.1-17.2)

Report productivity (RP): Normalises the output of staff to the daily report volume.

Note: SR = speech recognition ST = speech technology HT = human transcription RTT = report turnaround time WRR = word recognition rate PACS = picture archiving and communication system RP = report productivity TS = traditional transcription service ED = emergency department Sig. = Significant Diff = difference. TAT = turnaround time, equivalent to RTT.

## Results

### Productivity

The search strategy yielded six studies that evaluated the effect of SR systems on productivity— report turnaround time (RTT), or proportions of documents completed within a specified time period. Overall, most papers [22, 29, 36–38] reported significant improvement in RTT with SR. Two studies reported a significant reduction of RTT when SR was used to generate patient notes in an emergency department (ED) setting [29] and clinical notes in endocrinology [22]. A longitudinal study (20,000 radiology examinations) indicated that using SR reduced RTTs by 81% with reports available within one hour increasing from 26% to 58% [36]. Similarly, the average RTT of surgical pathology reports was reduced from four days to three days with increases in the proportion of reports completed within one day (22% to 36%) [23]. Zick and Olsen reported the reduction in RTT achieved by using SR in ED resulted in annual savings of approximately $334,000 [38].

Results of another study reported significant differences in RTT between SR systems produced by different companies. The authors reported that Dragon software took the shortest time (12.2 mins) to dictate a 938-word discharge report followed by IBM and L & H [33].

### Quality of reports

The quality of the reports in seven studies was determined by comparing errors or accuracy rates [18, 23, 29, 30, 33, 35, 38]. Taken together results from these studies suggest that human transcription is slightly more accurate than SR. The highest reported average accuracy rate across the included studies was 99.6% for human transcription [18] compared to 98.5% for SR [38]. However, an ED study found that reports generated by SR did not have grammatical errors while typed reports contained spelling and punctuation mistakes [29].

Evidence from the included studies also suggests that error rates are dependent on the type of SR system. A comparison of three SR systems indicated that IBM ViaVoice 98 General Medical Vocabulary had the lowest overall error rates compared with Dragon Naturally Speaking Medical Suite and L&H Voice X-press for Medicine, General Medicine Edition, when used for generating medical record entries [33]. A similar comparative analysis of four dental SR applications reported variation with regards to: time required to complete training, error rates, total number of commands required to complete specific tasks, dental specific functionality, and user satisfaction [34].

### System design

Some SR systems incorporated generic templates and dictation macros that included sections for specific assessment information such as chief complaint, history of present illness, past medical history, medications, allergies and physical examination [22, 38]. Other researchers used SR systems with supplementary accessories for managing text information such as generic templates [22], medical or pathology terminology dictionary [18, 20, 33, 38], Radiology Information System (RIS) [37] and Picture Archiving and Communication System (PACS) [36]. Evidence from these studies suggests that the use of additional applications such as macros and templates can substantially improve turnaround times, accuracy and completeness of documents generated using SR.

## Discussion

The purpose of this review was to provide contemporary evidence on SR systems and their application within health care. From this review and within the limitations of the quality of the studies included, we suggest that an SR system can be successfully implemented in a variety of health care settings with some considerations.

Several studies compared the use of transcribers to SR with human transcription having slightly higher overall word accuracy [18, 22, 36, 38] although with increased grammatical errors [29]. SR, although not as accurate (98.5% SR, 99.7% transcription [38]) with 10.3% to 15.2% error rates [33, 35], does deliver other benefits. Significantly improved patient outcomes such as reduced turnaround times for reporting [20, 23, 36–38] and cost-effectiveness [20, 38] have been demonstrated, however, equivocal evidence exists on improved workflow processes with Derman and colleagues finding no significant improvement [32].

Several issues related to the practical implementation of SR systems have been identified.

As with any information system [39], a SR system represents the interplay of staff, system, environment, and processes. A diverse range of health professionals and support staff were included in these studies with no demonstrable differences in training or accuracy, however typists (including health professionals) who are competent and presumably fast typists have some difficulty adapting to SR systems [22] ie., more benefit is obtained for slower typists. Also the length of transcription does seem to raise some concerns with text of 3 minutes or less recording time being problematic [22]. The nature of the information to be transcribed is also important as repetitive clinical cases frequently seen in settings such as radiology [36] or the emergency department [29], where templates or macros are easily adapted to the setting, are more likely to succeed. Applications relating to the writing of progress notes within psychiatry were limited in their success suggesting that other approaches or advances may be required where opportunities for standardised information is reduced [40].

In the majority of the included studies the reported error rates and improvements and other outcomes were achieved after only limited training was provided to participants who had no prior experience with SR. Training delivered varied from 5 minutes [29] to 6 hours [22], but several researchers advised that either a pre-training period using any speech recognition system [22] for one month or prolonged exposure with SR (one to three months) [20] is preferred. This is confirmed by the improved turnaround times demonstrated in longitudinal studies [36].

Technical aspects of system selection, vocabulary applied, and the management of background noise and accented voices are all challenges during implementation. System selection is important with several systems available with varying levels of recognition errors (7.0%-9.1% IBM ViaVoice98 General Medicine Vocabulary to 14.1%-15.2% L&H Voice Xpress for Medicine General medicine Edition) [33], but with nonetheless relatively low error rates. Dawson and colleagues [41] noted that nurses expectations of the accuracy of speech recognition systems were low.

Accuracy also varies depending upon the vocabulary used with potential users needing to consider the appropriate vocabulary for the task— using a pathology vocabulary [18], and using a general medicine vocabulary [33]—to minimise recognition errors. For example laboratory studies varying vocabularies for nursing handover confirmed that using the nursing vocabulary was more accurate than using the general medical vocabulary in the Dragon Medical version 11.0 (72.5% vs. 57.1%) [42].

Most contemporary SR systems have advanced microphones that have noise cancelling capacities that allow for SR systems to be used in noisy clinical environments [18, 30].

SR systems now accommodate some accented voices such as Dragon Medical™ providing accented voice profiles, for Australian English, Indian English and South East Asian English [40]. Finally the use of standardised terminology is recommended such as *the Voice Recognition Accuracy standards- by the National Institutes of Standards and Technology* [22] when reporting study outcomes.

### Limitations of the study

Whereas every endeavour was made to optimise inclusivity, the heterogeneity of the studies made comparative analysis and synthesis of the data challenging. The studies included in this review represent comparative designs or descriptive evaluations and only further rigorous clinical trials can confirm or refute the findings proposed here. A thorough examination of the cost benefits of SR in specific clinical settings needs to be undertaken to confirm some of the economic outcomes proposed or demonstrated here. The focus on patient turnaround times in reporting of radiographic procedures or assessment within the emergency department has the potential to increase patient flow and reduce waiting times. Additionally, SR has the potential to automatically generate standardised, terminology-coded clinical records and dynamically interact with clinical information systems to enhance clinical decision-making and improve time-to-diagnosis. Taking into account these areas in future evaluations will allow for a more comprehensive assessment of the overall impact that SR systems can have on quality of care and patient safety, as well as efficiency of clinical practice. We acknowledge the importance of publication bias relating to non-publication of studies or selective reporting of results that may affect the findings of this review.

## Conclusions

SR systems have substantial benefits but these benefits need to be considered in light of the cost of the SR system, training requirements, length of transcription task, potential use of macros and templates, and the presence of accented voices. The regularity of use enhances accuracy although frustration can result in disengaging with the technology before large accuracy gains are made. Expectations prior to implementation combined with the need for prolonged engagement with the technology are issues for management during the implementation phase. The improved turnaround times of patient diagnostic procedure reports or similar tasks represent an important outcome as it impacts on timely delivery of quality patient care. The ubiquitous nature of SR systems within other social contexts will guarantee improvements in SR systems (software and hardware). The availability of applications such as macros, templates, and medical dictionaries will increase accuracy and improve user acceptance. These advances will ultimately increase the uptake of SR systems by diverse health and support staff working within a range of healthcare settings.

## Authors’ information

MJ Faculty of Health Sciences Australian Catholic University, previously University of Western Sydney and Director, Centre for Applied Nursing Research (a joint facility of the South Western Sydney Local Health District and the University of Western Sydney), Sydney Australia. Affiliated with the Ingham Institute of Applied Medical Research.

SL Centre for Applied Nursing Research (a joint facility of the South Western Sydney Local Health District and the University of Western Sydney), Sydney Australia.

VL School of Computing, University of Western Sydney, Sydney, NSW, Australia.

PS University of Western Sydney, Sydney, NSW, Australia.

HS NICTA, The Australian National University, College of Engineering and Computer Science, University of Canberra, Faculty of Health, and University of Turku, Department of Information Technology, Canberra, ACT, Australia.

JB University of Western Sydney, Sydney, NSW, Australia.

LD University of Wollongong, Wollongong, NSW, Australia.

## Abbreviations

ChT: Charting time
ED: Emergency department
eHealth: Health informatics
HT: Human transcription
PACS: Picture achieving and communication systems
RCT: Randomised control trial
RIS: Radiology information system
RP: Report productivity
RTT: Report turnaround time
SCR: Speech contribution rates
SR: Speech recognition
ST: Speech technology
TT: Training time
WRR: Word recognition rate.

## References

[1] HISA: *Health Informatics Society of Australia*. http://www.hisa.org.au/. 2013 [cited 2014 14 January 2014]

[2] NEHTA:: *PCEHR*. http://www.nehta.gov.au/our-work/pcehr. 2014 [cited 2014 30th October 2014]

[3] Becker H (1972). Computerization of patho-histological findings in natural language. Pathol Eur.

[4] Anderson B, Bross IDJ, Sager N (1975). Grammatical compression in notes and records: analysis and computation. Am J Computational Linguistics.

[5] Hirschman L, Grishman R, Sager N (1976). From Text to Structured Information: Automatic Processing of Medical Reports. American Federation of Information Processing Societies: 1976.

[6] Collen MF (1978). Patient data acquisition. Med Instrum.

[7] Young DA (1982). Language and the brain: implications from new computer models. Med Hypotheses.

[8] Chi EC, Sager N, Tick LJ, Lyman MS (1983). Relational data base modelling of free-text medical narrative. Med Inform.

[9] Shapiro AR (1983). Exploratory analysis of the medical record. Medical Informatics Medecine et Informatique.

[10] Gabrieli ER, Speth DJ (1986). Automated analysis of the discharge summary. J Clin Comput.

[11] Mendonca EA, Haas J, Shagina L, Larson E, Friedman C (2005). Extracting information on pneumonia in infants using natural language processing of radiology reports. J Biomed Inform.

[12] Pakhomov SV, Buntrock JD, Chute CG (2006). Automating the assignment of diagnosis codes to patient encounters using example based and machine learning techniques. J Am Med Inform Assoc.

[13] Jamal A, McKenzie K, Clark M (2009). The impact of health information technology on the quality of medical and health care: a systematic review. HIM J.

[14] Kreps GL, Neuhauser L (2010). New directions in eHealth communication: opportunities and challenges. Patient Educ Couns.

[15] Waneka R, Spetz J (2010). Hospital information technology systems’ impact on nurses and nursing care. J Nurs Adm.

[16] Pearson JF, Brownstein CA, Brownstein JS (2011). Potential for electronic health records and online social networking to redefine medical research. Clin Chem.

[17] Suominen H (2012). The Proceedings of the Applications, and Resources for eHealth Document Analysis. CLEFeHealth2012 – the CLEF 2012 Workshop on Cross-Language Evaluation of Methods, Applications, and Resources for eHealth Document Analysis.

[18] Al-Aynati MM, Chorneyko KA (2003). Comparison of voice-automated transcription and human transcription in generating pathology reports. Arch Pathol Lab Med.

[19] Itakura F (1975). Minimum prediction residual principle applied to speech recognition. Acoustics, Speech and Signal Processing, IEEE Transactions on.

[20] Callaway EC, Sweet CF, Siegel E, Reiser JM, Beall DP (2002). Speech recognition interface to a hospital information system using a self-designed visual basic program: initial experience. J Digit Imaging.

[21] Houston JD, Rupp FW (2000). Experience with implementation of a radiology speech recognition system. J Digit Imaging.

[22] Mohr DN, Turner DW, Pond GR, Kamath JS, De Vos CB, Carpenter PC (2003). Speech recognition as a transcription aid: a randomized comparison with standard transcription. J Am Med Inform Assoc.

[23] Singh M, Pal TR (2011). Voice recognition technology implementation in surgical pathology: advantages and limitations. Arch Pathol Lab Med.

[24] Chaudhry B, Wang J, Wu S, Maglione M, Mojica W, Roth E, Morton S, Shekell PG (2006). Systematic review: impact of health information technology on quality, efficiency, and costs of medical care. Ann Intern Med.

[25] Goldzweig CL, Towfigh A, Maglione M, Shekelle PF (2009). Costs and benefits of health information technology: new trends from the literature. Health Aff.

[26] Buntin MB, Burke MF, Hoaglin MC, Blumenthal D (2011). The benefits of health information technology: a review of the recent literature shows predominantly positive results. Health Aff.

[27] Jones SS, Rudin RS, Perry T, Shekelle PG (2014). Health information technology: an updated systematic review with a focus on meaningful use. Ann Intern Med.

[28] Pluye P, Gagnon MP, Griffiths F, Johnson-Lafleur J (2009). A scoring system for appraising mixed methods research, and concomitantly appraising qualitative, quantitative and mixed methods primary studies in Mixed Studies Reviews. Int J Nursing.

[29] Northern Sydney Local Health District (2012). Manly Emergency Department Voice Recognition Evaluation.

[30] Alapetite A (2008). Impact of noise and other factors on speech recognition in anaesthesia. Int J Med Inform.

[31] Alapetite A, Andersen HB, Hertzum M (2009). Acceptance of speech recognition by physicians: a survey of expectations, experiences, and social influence. Int J Human-Computer Studies.

[32] Derman YD, Arenovich T, Strauss J (2010). Speech recognition software and electronic psychiatric progress notes: physicians’ ratings and preferences. BMC Med Inform Decis Mak.

[33] Devine EG, Gaehde SA, Curtis AC (2000). Comparative evaluation of three continuous speech recognition software packages in the generation of medical reports. J Am Med Inform Assoc.

[34] Irwin YJ, Gagnon MP, Griffiths F, Johnson-Lafleur J (2007). Speech recognition in dental software systems: features and functionality. Med Info.

[35] Kanal KM, Hangiandreou NJ, Sykes AG, Eklund HE, Araoz PA, Leon JA, Erickson BJ (2001). Initial evaluation of a continuous speech recognition program for radiology. J Digit Imaging.

[36] Koivikko M, Kauppinen T, Ahovuo J (2008). Improvement of report workflow and productivity using speech recognition a follow-up study. J Digit Imaging.

[37] Langer SG (2002). Impact of speech recognition on radiologist productivity. J Digital Imaging.

[38] Zick RG, Olsen J (2001). Voice recognition software versus a traditional transcription service for physician charting in the ED. Am J Emerg Med.

[39] Avison D, Fitzgerald G (2006). Information Systems Development: Methodologies, Techniques and Tools.

[40] Johnson M, Sanchez P, Suominen H, Basilakis J, Dawson L, Kelly B, Hanlen L (2014). Comparing nursing handover and documentation: forming one set of patient information. Int Nurs Rev.

[41] Dawson L, Johnson M, Suominen H, Basilakis J, Sanchez P, Estival D, Hanlen L (2014). A usability framework for speech recognition technologies in clinical handover: a pre-implementation study. J Med Syst.

[42] Suominen H, Ferraro G (2013). Noise in Speech-to-Text Voice: Analysis of Errors and Feasibility of Phonetic Similarity for Their Correction. Australasian Language Technology Association Workshop 2013.

[CR43] The pre-publication history for this paper can be accessed here:http://www.biomedcentral.com/1472-6947/14/94/prepub

